# Dose atrophy of vastus medialis obliquus and vastus lateralis exist in patients with patellofemoral pain syndrome

**DOI:** 10.1186/s13018-021-02251-6

**Published:** 2021-02-10

**Authors:** Conglei Dong, Ming Li, Kuo Hao, Chao Zhao, Kang Piao, Wei Lin, Chongyi Fan, Yingzhen Niu, Wang Fei

**Affiliations:** Department of Orthopaedic Surgery, Third Hospital of Hebei Medical University, Shijiazhuang, 050051 Hebei China

**Keywords:** Vastus medialis obliquus, Vastus lateralis muscle, Computed tomography, Patellofemoral pain syndrome, VMO/VLM area ratio

## Abstract

**Background:**

Whether vastus medialis obliquus atrophy exists in patients with patellofemoral pain syndrome and whether the amount of atrophy differs between the vastus medialis obliquus and vastus lateralis muscles remain unknown.

**Materials:**

From June 2016 to March 2019, 61 patients with patellofemoral pain syndrome were retrospectively included in the study group, and an age-, sex-, and body mass index-matched cohort of 61 patients with normal knees was randomly selected as the control group. All enrolled subjects had undergone CT scans in the supine position. The cross-sectional areas of the vastus medialis obliquus and the vastus lateralis muscle in the sections 0, 5, 10, 15, and 20 mm above the upper pole of the patella were measured, and the vastus medialis obliquus/vastus lateralis muscle area ratio was evaluated.

**Results:**

In the study group, the vastus medialis obliquus areas and the vastus lateralis muscle areas in the sections that were 0, 5, 10, 15, and 20 mm above the upper pole of the patella were significantly smaller than the respective areas in the control group (*P* < 0.05). The vastus medialis obliquus/vastus lateralis muscle area ratio was significantly smaller at the upper pole of the patella (the section 0 mm above the upper pole of the patella) than the corresponding ratio in the control group (*P* < 0.05). No significant difference was noted between the two groups in the sections 5, 10, 15, and 20 mm above the upper pole of the patella (*P* > 0.05).

**Conclusion:**

In patients with patellofemoral pain syndrome, vastus medialis obliquus and vastus lateralis muscle atrophy existed in sections 0–20 mm above the upper pole of the patella, compared with normal controls, and atrophy of the vastus medialis obliquus was more evident than that of the vastus lateralis muscle at the upper pole of the patella. These findings support the rationale for the use of general quadriceps exercise combined with vastus medialis obliquus strengthening exercise as part of the rehabilitation programme for the patients with patellofemoral pain syndrome.

## Introduction

Patellofemoral pain syndrome (PFPS) is one of the most common musculoskeletal complaints and is characterized as pain in the anterior knee region when performing activities such as sitting, stair climbing, running, and squatting [1, 2]. The exact pathogenesis of PFPS has been proposed to be multifactorial, and one of the main suggested contributing factors is patellar malalignment or abnormal patellar instability [3].

The function and the stability of the patellofemoral joint are maintained by a complex interaction among the active stabilizers, passive stabilizers, and osseous and cartilage morphology [4–7]. The vastus medialis muscle (VMM), especially the vastus medialis obliquus (VMO), which is a dynamic medial soft tissue stabilizer, plays an important role in the stability of the patellofemoral joint [8–10].

The VMO was described as the distal portion of the VMM with the muscle fibres inserted at a 50° angle into the longitudinal patellar alignment. The structure of the VMO makes it potentially able to partially counterbalance the lateral pull of the vastus lateralis muscle (VLM) [11–13]. Studies have shown that the weakness of the VMO causes the patellar lateral shift at 0 and 15° of knee flexion and is correlated with patellofemoral pain syndrome [8, 10].

However, whether VMO atrophy exists in PFPS patients remains obscure. Doxey [14] showed that 28 of 49 participants with PFPS had quadriceps atrophy by measuring the thickness of the quadriceps. Kaya et al. [15] and Pattyn et al. [16] found that the cross-sectional area of the VMO in patients with PFPS was smaller than that on their asymptomatic side. However, Balcarek et al. [17] and Callaghan [18] reported no significant difference in the cross-sectional area of the VMO between knees with PFPS and normal knees.

In addition, the majority of the studies focusing on VMO overlook the change in VLM in patients with PFPS, which also decreases muscle strength. Therefore, we measured the cross-sectional area of the VMO and VLM in the sections 0, 5, 10, 15, and 20 mm above the upper pole of the patella on CT scans, and VMO/VLM area ratios were also evaluated.

The purpose of this study was to evaluate whether VMO and VLM atrophy exists in patients with PFPS and whether the amount of atrophy differs between VMO and VLM. It is hypothesized that VMO and VLM atrophy existed in the patients with PFPS, and atrophy of the VMO was more evident than that of the VLM.

## Materials and methods

### Participants

In the present study, 61 patients were retrospectively included in the study group. Our inclusion criteria were as follows: (1) patients treated at the Third Hospital of Hebei Medical University from June 2016 to March 2019; (2) patients aged from 18 to 45 years (to avoid the influence of developmental factors and the likelihood of osteoarthritic changes in the patellofemoral joint); (3) patients who underwent CT scan; (4) anterior knee pain provoked by at least 2 of the following activities: prolonged sitting with flexed knees, stair climbing, squatting, running, kneeling, and jumping; (5) intermittent or continuous pain that persisted for more than 3 months; and (6) patients exhibiting 2 or more of the following clinical criteria on assessment: pain on direct compression of the patella against the femoral condyles with the knee in full extension, tenderness on palpation of the posterior edge at the medial and/or lateral border of the patella, pain on resisted knee extension, and pain on direct compression of the patella against the femur during isometric quadriceps contraction with the knee in slight flexion [1]. All the patients underwent Kujala scoring to assess their pain, and the average Kujala score was 73.22 (ranged from 69 to 82).

The exclusion criteria were as follows: (1) a period of non-weight bearing or any internal knee derangement due to a previous knee surgery or injury; (2) other knee disorders such as fracture, ligament injury, or meniscal injury; and (3) patellofemoral arthritis greater than grade II, where the patellofemoral joint surface represented a bony contact (Iwano classification) [19]. Two patients were excluded from the study group due to the previous knee surgery.

The control group which was matched with the experimental group according to sex, age, and body mass index (BMI) included 61 subjects without a history of patellofemoral joint-related diseases.

### CT protocols

All patients underwent CT examination in the supine position, with the knee fully extended, and the quadriceps muscles relaxed. The limbs were fixed by equipment to minimize motion. All examinations were performed with the same CT scanner (SOMATOM Sensation 16; Siemens Medical Solutions, Erlangen, Germany). The CT scanning parameters included a tube voltage of 120 kV, 100 effective mAs, 1-mm slice thickness, a gantry rotation time of 1 s, and a matrix size of 512 × 512. All measurements were performed using RadiAnt DICOM software (Medical Ltd., Poznan, Poland).

### Assessment

All patients obtained CT images of the hip and knee to measure the cross-sectional area of the VMO and VML, and the measurement was obtained using the annotation tool of the picture archiving and communication system (PACS) workstation (Centricity, GE Healthcare, St. Gilles, UK).

**Fig. 1:**
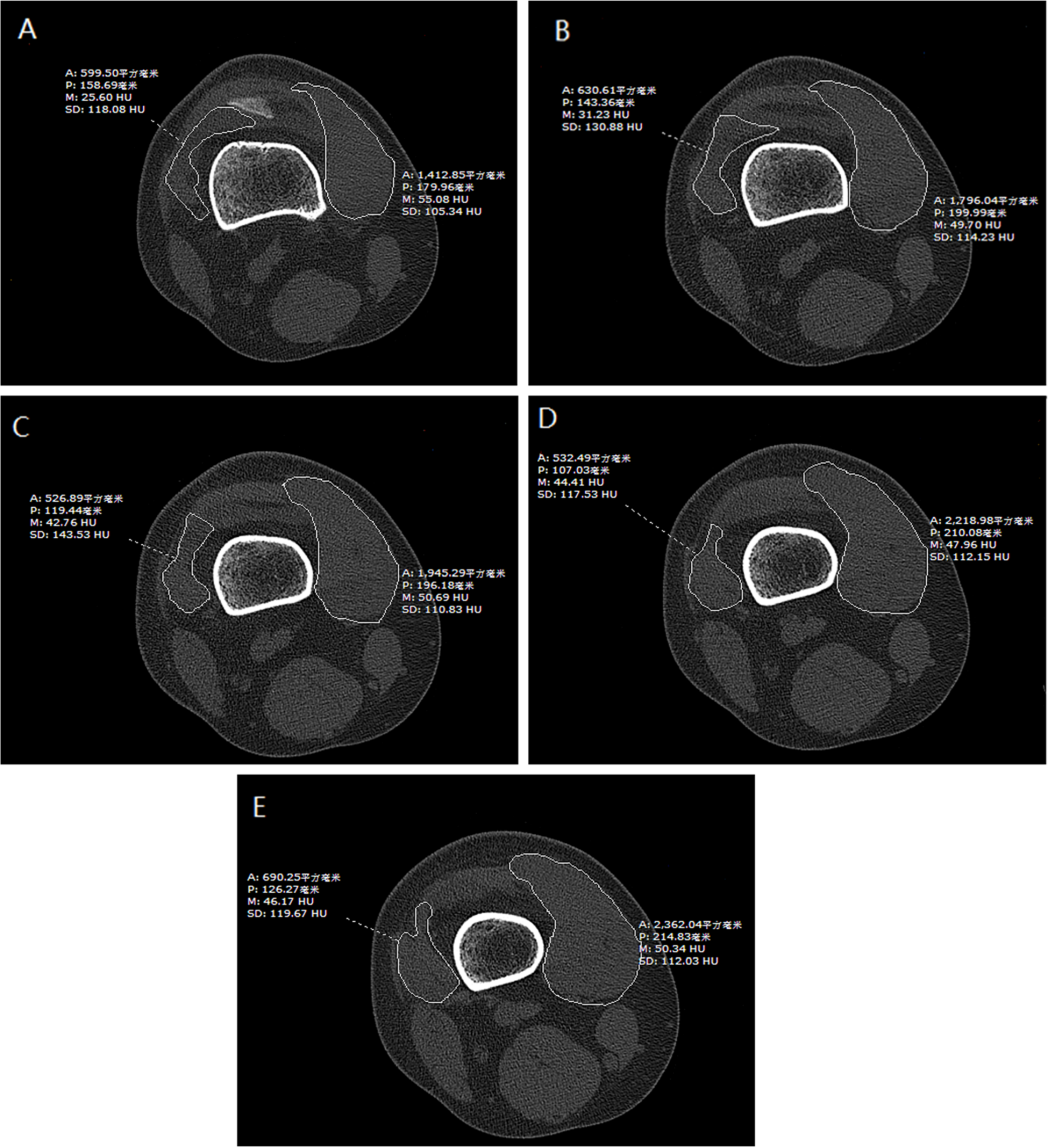
**a**–**e** Measurement of the cross-sectional area of the VMO and VLM in the section that 0–20 mm above the upper pole of the patella

First, we ensured that the scans of the hip and the knee were in the same position. Second, we defined the sections that were 0, 5, 10, 15, and 20 mm above the upper pole of the patella and measured the cross-sectional area by manually drawing contours around the muscle boundaries using two trained observers (Dong and Li) in each section (Fig. 1).

Our measurement methods had an accuracy of 0.01 mm^2^. The 2 observers were blinded to the characteristics of the patients and obtained all measurements independently. Intraclass correlation coefficient values (ICCs) were calculated to test intra- and inter-observer reliability.

### Statistical analysis

We used SPSS statistical software (version 21.0; SPSS Inc., Chicago, IL, USA) for statistical analyses. The VMO/VLM area ratio and the cross-sectional area of the VMO and VLM were evaluated using the Student paired *t* test. *P* values less than 0.05 were defined as significantly different.

## Results

In this study, all data are expressed as the mean ± standard deviation. No significant differences in BMI or age were noted between the study group and the control group. The demographics of the patients are summarized in Table 1. The intra-rater reliability was excellent for all the measurements, and the inter-rater reliability was high (Table 2).

**Table 1:** The demographics of the patients

	Numbers	Age (years)	Male/female	Side (left/right)	BMI
Study group	61	25.03 ± 5.94 (18–37)	30/ 31	27/34	23.76 ± 4.65
Control group	61	21.85 ± 7.40 (18–45)	37/ 24	32/29	26.08 ± 4.37
*P* value	/	*< 0.05*	/	/	*< 0.05*

*BMI* body mass index

**Table 2:** Intraclass correlation coefficients

		Intratester reliability ICC (95% CI)	Intertester reliability ICC (95% CI)
Study group	VMO areas	0.98 ^b^ (0.95–0.99)	0.96 ^b^ (0.91–0.98)
	VML areas	0.97 ^b^ (0.94–0.99)	0.95 ^b^ (0.90–0.97)
Control group	VMO areas	0.99 ^b^ (0.98–0.99)	0.98 ^b^ (0.96–0.99)
	VML areas	0.98 ^b^ (0.95–0.99)	0.96 ^b^ (0.92–0.98)

*ICC* intraclass correlation coefficient, *CI* confidence interval, *VMO* vastus medialis obliquus, *VLM* vastus lateralis muscle

^b^*P* < 0.001

In the study group, the vastus medialis obliquus areas and the vastus lateralis muscle areas in the section that were 0, 5, 10, 15, and 20 mm above the upper pole of the patella were significantly smaller than the respective areas in the control group (*P* < 0.05). The vastus medialis obliquus/vastus lateralis muscle area ratio was significantly smaller at the upper pole of the patella (the section 0 mm above the upper pole of the patella) than the corresponding area in the control group (*P* < 0.05), and there was no significant difference between the two groups in the sections 5, 10, 15, and 20 mm above the upper pole of the patella (*P* > 0.05) (Table 3).

**Table 3:** The cross-sectional area of the VMO, VLM, and the ratio of the cross-sectional area of the VMO to VLM in different sections in different sections

Group VMO ,VLM area, and the area ratio Sections	The upper pole of the patella (mm^2^)	5 mm above the upper pole of the patella (mm^2^)	10 mm above the upper pole of the patella (mm^2^)	15 mm above the upper pole of the patella (mm^2^)	20 mm above the upper pole of the patella (mm^2^)
The cross-sectional area of the VMO in different sections					
Study group	732.64 ± 306.43	876.32 ± 341.47	1039.31 ± 410.21	1178.26 ± 449.10	1289.78 ± 487.78
Control group	941.66 ± 366.83	1119.16 ± 405.01	1302.75 ± 425.14	1496.67 ± 474.70	1643.33 ± 507.08
*P* value	*< 0.05*	*< 0.05*	*< 0.05*	*< 0.05*	*< 0.05*
The cross-sectional area of the VLM in different sections					
Study group	127.61 ± 66.74	183.47 ± 85.41	250.66 ± 133.70	326.06 ± 139.94	413.27 ± 190.18
Control group	192.27 ± 152.40	262.55 ± 187.98	352.35 ± 291.96	446.22 ± 343.11	574.19 ± 390.00
*P* value	*< 0.05*	*< 0.05*	*< 0.05*	*< 0.05*	*< 0.05*
The ratio of the cross-sectional area of the VMO to VLM in different sections					
Study group	0.83 ± 0.11	5.37 ± 2.49	4.64 ± 2.43	3.90 ± 1.55	3.42 ± 1.36
Control group	7.44 ± 5.13	6.32 ± 4.69	4.15 ± 1.94	3.96 ± 1.66	3.48 ± 1.62
*P* value	*< 0.05*	*> 0.05*	*> 0.05*	*> 0.05*	*> 0.05*

*VMO* vastus medialis obliquus, *VLM* vastus lateralis muscle

## Discussion

The main findings of this study showed that VMO and VLM atrophy existed in the sections 0–20 mm above the upper pole of the patella in the PFPS patients, and the atrophy of the VMO was more evident than that of the VLM at the upper pole of the patella. These findings support the rationale for the use of general quadriceps exercise with VMO strengthening exercise as part of a rehabilitation programme for the patients with PFPS. To the best of our knowledge, this is the first study to evaluate the cross-sectional areas of the VMO and VLM and their ratio between normal controls and the patients with PFPS on CT scans.

Of note, as a dynamic medial soft tissue stabilizer, VMO plays an important role in the stability of the patellofemoral joint, and this role is attributed to its special structure. The VMO has distal muscle insertion that is a 50° angle to the longitudinal patellar alignment and also has the strong meshing fibres with the medial patellofemoral ligament near its distal insertion [12, 13, 20].

Studies have shown that in patients complaining of PFPS, the quadriceps exhibit weakness [10, 21]. Although it is well known that maximum strength is related to muscle size [22], whether the VMO atrophy exists in PFPS remains controversial [14–18].

Three measurements were used to evaluate the atrophy of the VMO: tape measurement, ultrasound, and MR imaging. In the clinical setting, girth measurements with tape are the most common estimations of quadriceps atrophy, but this method involves other thigh muscles as well as bone and subcutaneous fat. MR imaging is the “gold standard” of muscular measurement, and the mean difference between ultrasound and MR imaging is only 0.8% [18].

Similar to MRI, CT is also considered a highly precise imaging modality for investigating the area and volume of muscle and has a reported precision error of approximately 1.4% for tissue areas, both scanning methods are able to distinguish muscle mass from fat [23]. In the present study, we first selected CT as our measurement method, and we found that the cross-sectional area of the VMO was significantly smaller in the patients with PFPS compared with normal control. VMO atrophy certainly existed in this population.

In addition to the VMO, the VLM in the subjects with PFPS had decreased muscle strength according to electromyography [10]. However, literature comparing the size of the VMO relative to the VML between PFPS and asymptomatic limbs is lacking. Only Giles et al. [24] and Pattyn et al. [16] reported that selective atrophy of the VMO relative to the VLM was not identified in people with PFP using ultrasound and MR imaging, respectively. However, Giles et al. did not measure the cross-sectional area of the muscle but the thickness, and Pattyn et al. only measured the VMO/VLM area ratio on the patellar level and mid-thigh level.

In the present study, we found that VLM atrophy existed in PFPS patients, and atrophy of VMO was more evident than that of VLM at the upper pole of the patella. The distal portion is the main functional area of the VMO to confine the patellar maltracking, and obvious VMO atrophy must influence the patellar stability.

The finding of the VMO and VML atrophy, especially the distal insertion of the VMO in subjects with PFPS, contributes to understanding the mechanisms of PFPS [10, 21]. Decreased quadriceps weakness that results from atrophy or pain limiting force production, pain-induced inhibition of the quadriceps musculature, or physiological changes of the quadriceps musculature has been suggested as a potential cause of PFPS [15, 18, 25]. Although we could not determine whether atrophy was a predisposing factor or developed after the onset of PFPS, given the existence of the VMO and VLM atrophy, physiotherapy with strengthening of the quadriceps must be beneficial for patients to restore quadriceps strength and relieve pain [26].

The isolated VMO activation protocol has been used to treat patellofemoral pain and instability, but Syme et al. [26] reported no difference between rehabilitation with selective VMO exercise and general quadriceps strengthening exercises. In the present study, we still suggested VMO strengthening exercise to patients with PFPS, given atrophy of the VMO, especially its distal portion. However, we should not overlook the contribution of the VLM and other muscles of the quadriceps to patellar stability, and general quadriceps exercise was also suggested. In conclusion, the protocol of general quadriceps exercise combined with VMO strengthening exercise may represent a better choice.

One of the limitations of this study is that the sample size was small, and the present study was a single-centre retrospective study, which could lead to bias. The CT examination is performed after patients complain of PFPS. Therefore, we cannot determine whether the change in VMO and VLM is the cause or result of PFPS.

## Conclusions

In the patients with PFPS, VMO and VLM atrophy existed in the section 0–20 mm above the upper pole of the patella in comparison with normal people, and VMO atrophy was more evident than that of the VLM at the upper pole of the patella. These findings support the rationale for the use of general quadriceps exercise with VMO strengthening exercise as a part of rehabilitation programme for patients with PFPS.

## Abbreviations

VMO: Vastus medialis obliquus
VLM: Vastus lateralis muscle
PFPS: Patellofemoral pain syndrome
BMI: Body mass index
CT: Computed tomography
